# Establishing a quality assurance program for photon counting detector (PCD) CT: Tips and caveats

**DOI:** 10.1002/acm2.14074

**Published:** 2023-06-19

**Authors:** Zaki Ahmed, Andrea Ferrero, Liqiang Ren, Thomas J. Vrieze, Kishore Rajendran, Christopher P. Favazza, Lifeng Yu, Michael R. Bruesewitz, Cynthia H. McCollough, Shuai Leng

**Affiliations:** ^1^ Department of Radiology Mayo Clinic Rochester Minnesota USA; ^2^ Department of Radiology William Beaumont University Hospital Royal Oak Michigan USA; ^3^ Department of Radiology UT Southwestern Medical Center Dallas Texas USA

**Keywords:** photon counting detector, quality assurance, quality control, scanner performance

## Abstract

**Purpose:**

To determine the suitability of a quality assurance (QA) program based on the American College of Radiology's (ACR) CT quality control (QC) manual to fully evaluate the unique capabilities of a clinical photon‐counting‐detector (PCD) CT system.

**Methods:**

A daily QA program was established to evaluate CT number accuracy and artifacts for both standard and ultra‐high‐resolution (UHR) scan modes. A complete system performance evaluation was conducted in accordance with the ACR CT QC manual by scanning the CT Accreditation Phantom with routine clinical protocols and reconstructing low‐energy‐threshold (T3D) and virtual monoenergetic images (VMIs) between 40 and 120 keV. Spatial resolution was evaluated by computing the modulation transfer function (MTF) for the UHR mode, and multi‐energy performance was evaluated by scanning a body phantom containing four iodine inserts with concentrations between 2 and 15 mg I/cc.

**Results:**

The daily QA program identified instances when the detector needed recalibration or replacement. CT number accuracy was impacted by image type: CT numbers at 70 keV VMI were within the acceptable range (defined for 120 kV). Other keV VMIs and the T3D reconstruction had at least one insert with CT number outside the acceptable range. The limiting resolution was nearly 40 lp/cm based on MTF measurements, which far exceeds the 12 lp/cm maximum capability of the ACR phantom. The CT numbers in the iodine inserts were accurate on all VMIs (3.8% average percentage error), while the iodine concentrations had an average root mean squared error of 0.3 mg I/cc.

**Conclusion:**

Protocols and parameters must be properly selected on PCD‐CT to meet current accreditation requirements with the ACR CT phantom. Use of the 70 keV VMI allowed passing all tests prescribed in the ACR CT manual. Additional evaluations such an MTF measurement and multi‐energy phantom scans are also recommended to comprehensively evaluate PCD‐CT scanner performance.

## 1 INTRODUCTION

Recent advances in photon counting detector (PCD) technology have resulted in the first whole‐body commercial PCD‐CT scanner (NAEOTOM Alpha; Siemens Healthineers, Forchheim, Germany) being cleared by the FDA for clinical use in 2021.^1^, ^2^, ^3^, ^4^, ^5^ Like any other clinical CT system, a rigorous quality assurance program is necessary to ensure safe and effective diagnostic use of the system.^6^ There are several features of PCDs that may limit the direct translation of quality control (QC) procedures that were designed for conventional CT systems built on energy‐integrating‐detectors (EIDs). Specifically, PCDs weigh each detected photon the same irrespective of its energy, whereas EIDs weigh the detected photons proportionally to their deposited energy.^7^, ^8^ As a result, PCD‐CT generates images with a lower effective energy than their EID‐CT counterparts, resulting in different CT numbers for the same materials, even if the acquisition and reconstruction parameters are similar. Therefore, acceptable CT number ranges selected for EID‐CT systems may not be valid for PCD‐CT systems. Additionally, PCDs can be manufactured with a smaller form factor than EIDs without a loss of geometric efficiency, as they do not need septa materials to prevent visible light from crossing over to neighboring detector pixels.^9^, ^10^ As a result, PCD‐CT systems can achieve higher spatial resolution than EID‐CT. Therefore, the range of clinical spatial resolution assessed with conventional phantoms designed for EID‐CT may no longer be suitable to characterize certain PCD‐CT clinical protocols. Finally, PCD provides intrinsic multi‐energy information by means of two or more energy thresholds applied to the detected photons.^11^, ^12^, ^13^ This capability and the need to assess overall detector stability may require changes to the number and frequency of QC tests performed to assess spectral performance. Our purpose in this study was to determine the suitability of a quality assurance (QA) program based on the American College of Radiology's (ACR) CT QC manual to fully evaluate the unique capabilities of a clinical dual‐source PCD‐CT system.

## 2 METHODS

The first commercial, whole‐body, PCD‐CT (NAEOTOM Alpha; Siemens Healthineers) was used throughout this work. Whenever comparisons with an EID‐CT system are provided, images were obtained on a third‐generation dual‐source EID‐CT system from the same manufacturer (SOMATOM Force; Siemens Healthineers).

### 2.1 Daily QA program

The ACR recommends a daily CT quality control procedure be performed by the technologist prior to the first clinical scan of the day. The objectives of the QA procedure were to (a) ensure stable calibration of CT numbers relative to water, (b) ensure stable image noise, and (c) identify any image artifacts before patient scanning. A single scan, helical or axial, of a uniform phantom, is typically performed on EID‐CT systems as part of this procedure. The PCD‐CT system has two separate source‐detector subsystems (A and B) and two main acquisition modes: standard mode with 144 × 0.4 mm collimation and ultra‐high‐resolution (UHR) mode with 120 × 0.2 mm collimation. Additionally, different energy thresholds are automatically selected by the system depending on the chosen tube potential. Because PCDs require more careful thermal equilibrium for their optimal operation and lack the decades of clinical experience of their EID counterparts, we adapted our daily QC procedure to include 4 scans of a uniform phantom, all with 120 kV:

1. An axial scan in standard mode with subsystem A;
2. A helical scan in standard mode with subsystem A;
3. A helical scan in UHR mode with subsystem A;
4. A helical scan in standard mode with both subsystems (A&B).

All scans were reconstructed to generate low‐energy threshold (named T3D on the scanner) images that include all detected photons. The slice thickness was set to 5 mm to reduce the number of images the technologist was required to review. The noise limits were selected for each series based on 90 days’ worth of daily QC scans, as is standard in our practice. For artifact visualization, we followed the ACR QC manual recommendation and displayed the images with a 100/0 HU display setting for window width/center.

QC images were retrospectively reviewed around the dates of every major service intervention to the PCD‐CT system (e.g., detector replacement) to confirm (a) the identification of an issue affecting the clinical performance of the system and (b) its resolution following the repair.

### 2.2 Annual CT equipment performance evaluation

A complete annual system performance evaluation was conducted in accordance with the ACR CT QC manual by scanning the ACR CT Accreditation Phantom (CTAP) on both an EID‐CT and a PCD‐CT system with the routine clinical head and body protocols in our practice. The main tests reported here are the following:

1. Module 1: CT number linearity at all available tube potentials. For the PCD‐CT system, the CT number linearity was measured on T3D images along with virtual monoenergetic images (VMIs) between 40 and 120 keV.
2. Module 1: Slice thickness
3. Module 2: Low contrast detectability
4. Module 3: Spatial uniformity
5. Module 4: High‐contrast spatial resolution

The details of our protocols are listed in Tables 1, 2, 3, 4, 5.

**TABLE 1:** Relevant acquisition and reconstruction parameters for the CT number linearity as a function of kV station.

	Siemens SOMATOM Force (EID‐CT)	Siemens NAEOTOM Alpha (PCD‐CT)
kV	70‐150 (steps of 10), Sn100, Sn150	70,90,120,140, Sn100, Sn140
Eff mAs	180	150
Collimation [mm]	96x0.6	144x0.4
Image Type	Single energy	Low‐energy threshold (T3D)
Slice Thickness [mm]	3	3
Reconstruction Kernel	Br44	Br44
Iterative Recon / Strength	ADMIRE / 3	QIR / 3

**TABLE 2:** Relevant acquisition and reconstruction parameters for ACR image quality tests for the routine abdominal adult protocol.

	Siemens SOMATOM Force (EID‐CT)	Siemens NAEOTOM Alpha (PCD‐CT)
kV	120	120
Eff mAs	180	130
CTDI (mGy)	12	10.3
Collimation (mm)	96x0.6	144x0.4
Image Type	Single energy	VMI 70 keV
Slice Thickness (mm)	3	3
Reconstruction Kernel	Br44	Br44
Iterative Recon / Strength	ADMIRE / 3	QIR / 3

**TABLE 3:** Relevant acquisition and reconstruction parameters for ACR image quality tests for the routine abdominal pediatric protocol.

	Siemens SOMATOM Force (EID‐CT)	Siemens NAEOTOM Alpha (PCD‐CT)
kV	120	90
Eff mAs	40	77
CTDI (mGy)	2.1	2.1
Collimation (mm)	96x0.6	144x0.4
Image Type	Single energy	VMI 70 keV
Slice Thickness (mm)	3	3
Reconstruction Kernel	Br40	Br40
Iterative Recon / Strength	ADMIRE / 3	QIR / 3

**TABLE 4:** Relevant acquisition and reconstruction parameters for ACR image quality tests for the routine head adult protocol.

	Siemens SOMATOM Force (EID‐CT)	Siemens NAEOTOM Alpha (PCD‐CT)
kV	120	120
Eff mAs	250	200
CTDI (mGy)	35.7	33.8
Collimation (mm)	96x0.6	144x0.4
Image Type	Single energy	VMI 70 keV
Slice Thickness (mm)	5	5
Reconstruction Kernel	Hr40/Hr69	Hr40/Hr68
Iterative Recon / Strength	ADMIRE / 3	QIR / 3

**TABLE 5:** Relevant acquisition and reconstruction parameters for ACR image quality tests for the routine head pediatric protocol.

	Siemens SOMATOM Force (EID‐CT)	Siemens NAEOTOM Alpha (PCD‐CT)
kV	120	90
Eff mAs	130	300
CTDI (mGy)	18.6	24.4
Collimation (mm)	96x0.6	144x0.4
Image Type	Single energy	VMI 70 keV
Slice Thickness (mm)	3	5
Reconstruction Kernel	Hr40	Hr40
Iterative Recon / Strength	ADMIRE / 3	QIR / 3

**TABLE 6:** Relevant acquisition and reconstruction parameters for the additional characterization of spatial resolution and multi‐energy performance of the PCD‐CT system.

	MTF phantom (25 μm thick tungsten wire)	Multi‐energy phantom (Gammex body)
kV	120	120
Eff mAs	400	218
CTDI (mGy)	32	18
Collimation (mm)	120 x 0.2	144 x 0.4
Image Type	Single energy	VMIs 40 to 120 keV, iodine image
Slice Thickness (mm)	0.4	3
Reconstruction Kernel	Br96	Qr40
Iterative Recon / Strength	QIR / 3	QIR / 3

### 2.3 Additional tests

Additional measurements were performed to assess the spatial resolution and multi‐energy capabilities of the PCD‐CT. The limiting in‐plane resolution was evaluated by scanning a tungsten wire (50 μm diameter) with a sharp kernel (Br96) using the UHR acquisition mode, from which the modulation transfer function (MTF) was computed. The multi‐energy capability was evaluated by scanning a multi‐energy CT phantom (40 × 30 cm^2^; Sun Nuclear) containing four solid inserts with iodine concentrations of 2, 5, 10, and 15 mg L/cc and reconstructing an iodine map along with VMIs between 40 and 120 keV. The details of the acquisition and reconstruction protocols used for these two sets of tests are reported in Table 6. The values from the iodine map and VMIs were compared against reference values provided by the phantom manufacturer.

## 3 RESULTS

### 3.1 Daily CT QA program

**FIGURE 1:**
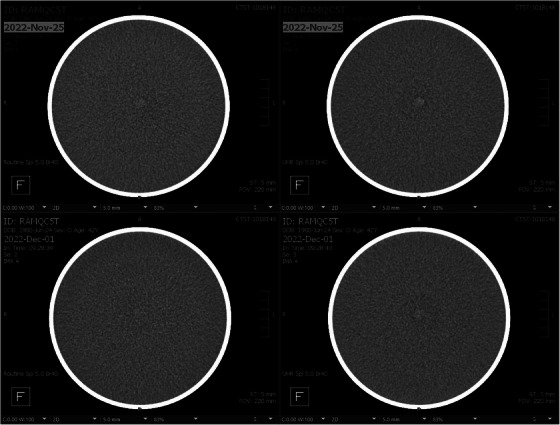
Daily CT QC images from the PCD‐CT system. Top row, initial data acquired in spiral mode with the standard (144 × 04 mm; left) and UHR (120 × 0.2 mm; right) detector configuration. Hyperdense artifact at the isocenter was noted, most prominent in the UHR mode, which contributed to the decision to replace detector modules during a scheduled system upgrade the following day. Bottom row, the same data acquired after the detector module replacement showed the artifact was strongly decreased. PCD,= photon‐counting‐detector; QC, quality control; UHR, ultra‐high‐resolution.

Sample daily CT QC images before and after a software upgrade that included detector module replacement are shown in Figure 1. The initial QC images (top row) show a clear center dot artifact, most visible in the UHR scan mode. The artifact was significantly reduced, and no longer of clinical concern, in the QC images acquired following detector replacement (bottom row).

### 3.2 Annual CT equipment performance evaluation

#### 3.2.1 Module 1 – CT number linearity

**FIGURE 2:**
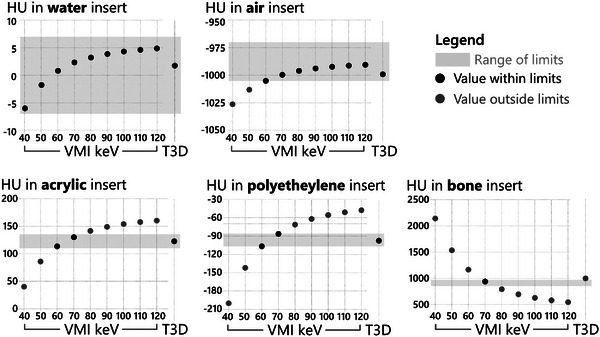
CT numbers (HU) of the 5 inserts measured at T3D and VMIs of 40 to 120 keV for the routine adult protocol. The shaded region represents the acceptable limits. Some inserts, such as water, are less sensitive to the VMI energy level and are within the ACR limits for all the tested VMIs. Meanwhile, some inserts, such as bone, are only within the ACR limits over a narrow keV range. Among the tested VMI energy levels, only the 70 keV VMI provides CT numbers that are within the ACR limits for all inserts. VMI, virtual monoenergetic images.

The first module of the ACR CTAP includes five inserts (Water, Air, Polyethylene, Acrylic, and Bone) and their CT numbers should fall within a prescribed range for a 120 kV scan. Tables A1 and A2 (Appendix) show the CT numbers measured for the Force and the Alpha scanners, respectively. For the EID‐CT data, all measurements were within the limits of the 120 kV single‐energy acquisitions. For the PCD‐CT data, the measured CT number for the bone insert was 1001 HU at 120 kV when using T3D images, which exceeds the upper limit of 970 HU in the ACR manual. On the other hand, all measurements were within the prescribed limits for the 70 keV VMI we used on the four routine clinical protocols. Other VMI reconstructions were also tested between 40 to 120 keV in steps of 10 keV/step, but only the 70 keV reconstruction was within limits for all inserts (Figure 2).

#### 3.2.2 Modules 2 and 3

**FIGURE 3:**
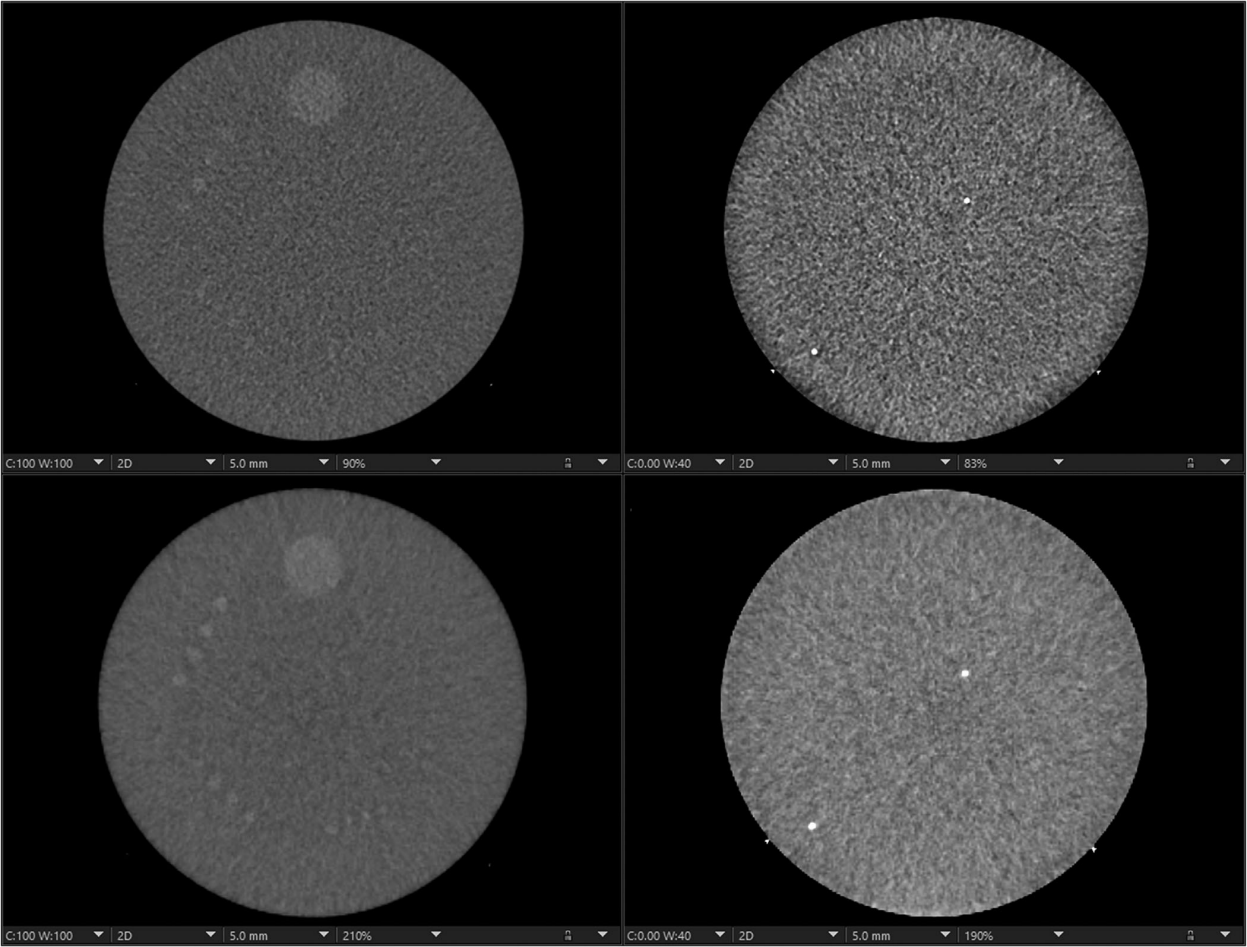
ACR CTAP images acquired with the routine adult head protocol. Top row, EID‐CT system (Force). Bottom row, PCD‐CT system (Alpha). Left column, Module 2, low contrast detectability. Right column, Module 3, image uniformity. CTAP, CT accreditation phantom; EID, energy integrating detector; PCD, photon counting detector.

There were no substantial differences between EID‐CT and PCD‐CT on the second and third modules of the ACR CTAP. In Figure 3, images of the central slice are shown for both modules and the EID‐CT and PCD‐CT scanners, using the routine adult head protocol. The performance is similar between the two systems, albeit the lower noise in the PCD‐CT images at matched spatial resolution (reconstruction kernel) allows for increased detectability of low‐contrast objects, such as the 5 and 4 mm inserts.

#### 3.2.3 Module 4 – Spatial resolution

**FIGURE 4:**
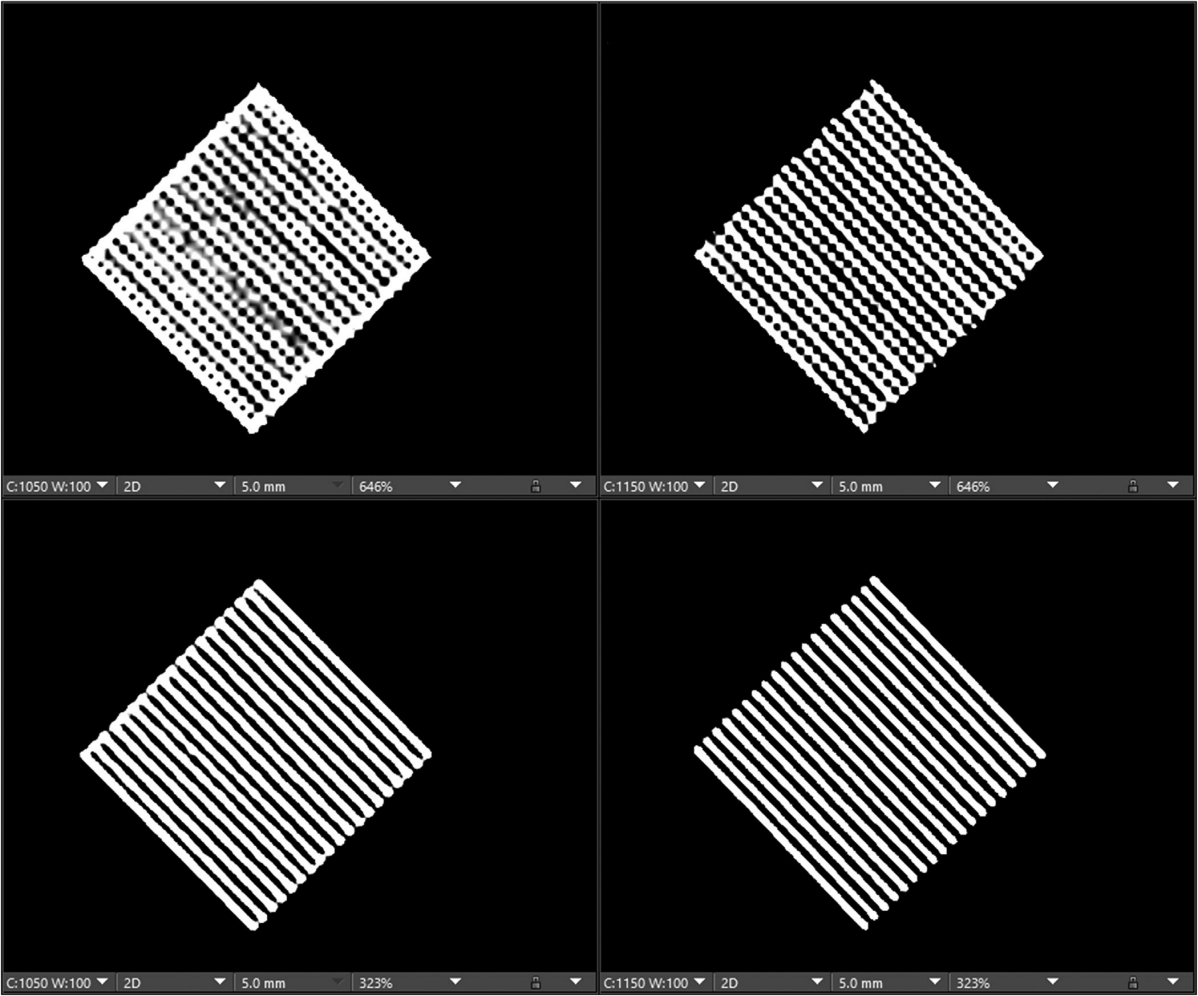
A magnified view of the 12 line‐pair per cm insert in the high‐contrast resolution portion of the CT accreditation phantom, as seen using a sharp kernel from the EID‐CT (left column) and PCD‐CT (right column). Top row: 512 matrix. Bottom row: 1024 matrix. Both scanners can resolve the insert, but the EID‐CT image shows discontinuities for the lines, especially with the smaller matrix size, while PCD‐CT does a better job at delineating the lines and is capable of achieving greater resolutions. EID, energy‐integrating‐detector; PCD, photon‐counting‐detector.

In Figure 4, we show a close‐up view of the highest resolution insert (12 lp/cm) for module 4 of the ACR CTAP phantom scanned on EID‐CT and PCD‐CT using the routine adult head protocol. Both the EID‐CT and PCD‐CT were able to resolve this insert with the typical bone reconstruction kernel, especially if higher resolution matrices (> 512) are available. However, the PCD‐CT has an additional ultra‐high‐resolution mode that can achieve even greater resolutions that significantly exceed the ACR CTAP.

### 3.3 Additional tests

**FIGURE 5:**
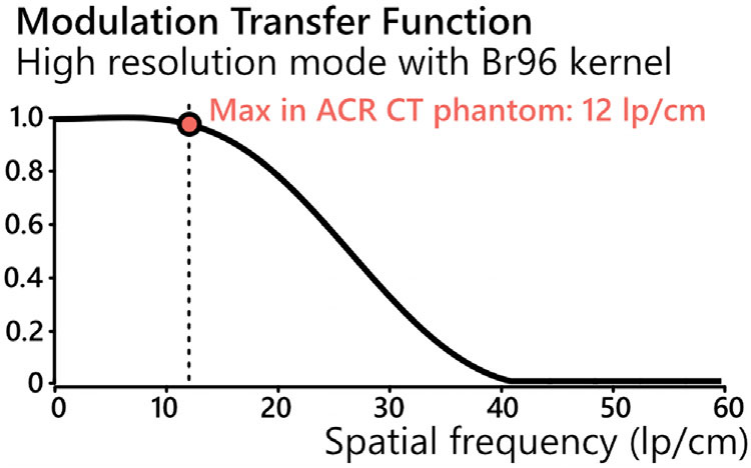
The modulation transfer function measured from the PCD‐CT using the ultra‐high‐resolution mode with a sharp kernel. The limiting resolution is near 40 lp/cm, which is far greater than the maximum resolution of 12 lp/cm in the ACR CT phantom. PCD , photon counting detector.

**FIGURE 6:**
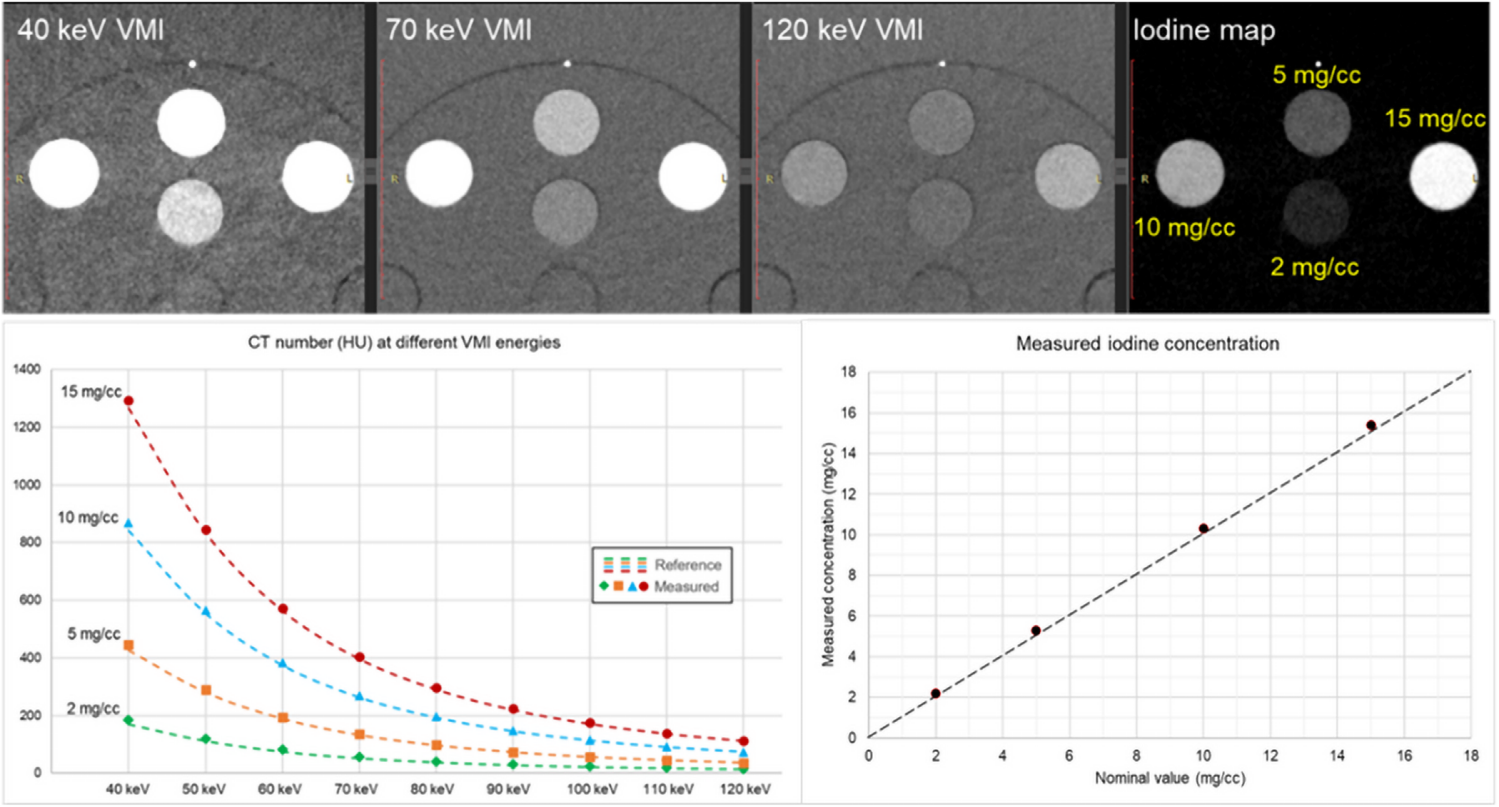
Sample images of the multi‐energy phantom at different VMIs along with the iodine map (top row). The iodine concentration of the four inserts is overlayed on the iodine map. The measured CT number in each insert (bottom left) was in good agreement (3.8% mean percent error) with the reference values provided by the phantom manufacturer. The concentrations measured in the iodine map (bottom right) were also in excellent agreement with the expected values for each insert, with measurements very close to the identity line. The RMSE between measured and nominal concentrations was 0.30 mgI/cc. RMSE, root mean squared error; VMI,= virtual monoenergetic images.

The spatial resolution of PCD‐CT's UHR mode was evaluated by computing the MTF curve and it indicated a limiting resolution of approximately 40 lp/cm (Figure 5). The multi‐energy capability was evaluated on a 40 cm phantom containing solid iodine inserts and the PCD‐CT provided accurate CT numbers on all VMIs with an average percentage error of 3.8% (Figure 6). Furthermore, the values on the iodine maps were accurate with an average root mean squared error of 0.3 mgI/cc (Figure 6).

## 4 DISCUSSION

The introduction of the first clinical PCD‐based CT system required some adjustments to the standard QC program that was designed based on EID‐CT, both in terms of the type of tests as well as passing criteria. In this work, we shared our initial experience customizing our QC program to account for the unique features of PCD‐CT.

Starting with the daily QC, we proposed and implemented changes to our protocol that evaluated the performance of the system in terms of CT number accuracy and artifacts for both standard and UHR acquisition modes and for both subsystems. This change to include the UHR acquisition mode was motivated by the expectation for a significantly increased prevalence of its use in routine clinical imaging in the body, owing to its improved dose efficiency compared to its EID‐CT counterpart.

As part of the annual CT performance evaluation, we focused our attention on exploring the challenges that the different photon weighting in PCD‐CT pose for the CT number linearity tests. Our approach was to rely on the low‐energy threshold images to assess the behavior of CT numbers for different tissue‐mimicking inserts as a function of tube potential. However, those images would not pass the current ACR limits for different tissue‐mimicking inserts using any of the clinical protocols as these limits were developed based on conventional EID‐CT. Rather than determining a customized set of limits for the PCD‐CT system, we opted to replace low‐energy threshold images with virtual monoenergetic images at 70 keV. This is due to the fact that the effective energy of the 120 kV spectrum on EID‐CT is close to 70 keV, which results in similar CT numbers between the 120 kV EID‐CT images and the 70 keV VMI on PCD‐CT. This allows the appropriate use of the ACR CT number limits on the PCD‐CT. With our approach, the PCD‐CT system passed all ACR tests for CT number linearity while simultaneously providing valuable information about beam quality at different kVs.

The ACR CT phantom tests were complemented with additional tests to further characterize the spatial resolution and multi‐energy capabilities of the PCD‐CT system in a quantitative manner. In particular, the MTF measurement was useful because not only can PCD‐CT achieve spatial resolutions of nearly 40 lp/cm, but also ultra‐high‐resolution is no longer limited to the extremities as previously on EID‐CT. As a result, several routine protocols that are typically assessed as part of the annual CT performance evaluation could now be scanned in this mode, which is capable of generating images with a spatial resolution that is far above the maximal 12 lp/cm of the ACR CT phantom. If the scanner spatial resolution performance were to substantially degrade—for example, following a collimator replacement or because of slight misalignment in the system optics, this would not be detected by the conventional ACR tests—but it would with the proper MTF tests. Similarly, the multi‐energy capability of the scanner is not adequately evaluated with the ACR phantom alone. Owing to the frequent software upgrades that are to be expected on a first‐of‐kind clinical system, it is our recommendation that multi‐energy phantom evaluation is benchmarked at acceptance testing and following major upgrades.^11^, ^12^, ^14^, ^15^ AAPM Task Group 299 is currently preparing a set of recommendations for routine QC of multi‐energy CT scanners.^16^ Although it is not specific to PCD‐CT, many recommended multi‐energy tests are directly applicable to PCD‐CT systems.

The additional tests proposed in the dedicated QA program for PCD‐CT scanners could have some time cost for both the technologist and the medical physicist. While we did not specifically quantify this time cost, as it is strongly dependent with user familiarity with the equipment, it is important to note that on the current clinical PCD‐CT scanner all multi‐energy reconstructions are generated and available at the scanner with virtually no delay compared to conventional reconstructions, and therefore the time cost is mainly limited to the interpretation and scoring of the additional series.

## 5 CONCLUSION

We shared our initial experience with establishing a QC program for a clinical PCD‐CT system. With proper modifications that account for the unique features of this system, it has been our experience that it is possible to successfully integrate such a system within established QA procedures designed for a fleet of EID‐CT scanners, without noticeable changes to the technologist workflow and without substantially increasing the time required for annual physics performance evaluations.

## AUTHOR CONTRIBUTIONS

Study concepts/study design or data acquisition or data analysis/interpretation: all authors; manuscript drafting or manuscript revision for important intellectual content: all authors; final approval of the submitted manuscript: all authors; agreement to be accountable for all aspects of the work in ensuring that questions related to the accuracy or integrity of any part of the work are appropriately investigated and resolved: all authors; literature research: Zaki Ahmed, Andrea Ferrero, Shuai Leng; experimental studies: Zaki Ahmed, Andrea Ferrero, Liqiang Ren, Thomas J. Vrieze, Kishore Rajendran, Michael R. Bruesewitz, Shuai Leng.

## CONFLICT OF INTEREST STATEMENT

Cynthia McCollough is the recipient of a research grant to the institution from Siemens Healthcare GmbH. The other authors have no relevant conflicts of interest to disclose.

Selected sections were presented during an oral presentation at the 2022 AAPM Annual Meeting.

## Appendix

**TABLE A1:** Recorded CT numbers using module 1 of the ACR CTAP phantom scanned on the EID‐CT.

kV		Poly −107 to −84 (ACR)	Water −7 to 7 (ACR)	Acrylic 110 to 135 (ACR)	Bone 850 to 970 (ACR)	Air −1005 to −970 (ACR)
70		−134.5	2.1	99.5	1366.5	−992.5
80		−118.1	1.0	109.8	1206.1	−996.2
90		−107.8	1.1	115.0	1100.1	−997.8
100		−99.5	1.9	122.0	1020.9	−998.1
110		−94.9	0.8	124.8	955.0	−998.1
120		−89.3	0.7	128.9	905.9	−998.2
130		−85.8	1.1	131.4	866.1	−998.5
140		−82.6	0.2	133.5	833.2	−998.0
150		−80.6	0.4	133.8	804.4	−998.2
Sn100		−76.7	1.7	136.5	824.8	−994.5
Sn150		−62.8	1.3	145.5	654.8	−995.7
DE 90 kV		−109.4	1.0	115.8	1101.3	−998.8
DE Sn150kV		−61.9	1.6	147.5	661.1	−998.7
120	(adult abd)*	−89.3	0.7	128.9	905.9	−998.2
120	(peds abd)*	−88.4	0.4	128.3	900.3	−1002.8
120	(adult head)*	−91.1	−0.8	128.8	927.8	−997.9
120	(peds head)*	−90.2	−1.0	129.2	929.5	−998.7

Asterisks indicate clinical protocols. Listed tolerances only apply to 120 kV for materials other than water and air.

**TABLE A2:** Recorded CT numbers using module 1 of the ACR CTAP phantom scanned on the PCD‐CT.

kV		Poly −107 to −84 (ACR)	Water −7 to 7 (ACR)	Acrylic 110 to 135 (ACR)	Bone 850 to 970 (ACR)	Air −1005 to −970 (ACR)
70		−136.3	2.7	99.0	1402.6	−997.5
90		−113.9	0.6	113.6	1150.5	−1000.7
120		−99.2	1.1	122.0	1000.8	−997.3
140		−93.9	1.3	126.0	942.1	−995.6
Sn100		−88.0		129.0	893.0	−994.0
Sn140		−74.0	−0.9	136.7	753.4	−991.5
UHR 120 kV		−89.5	2.1	130.3	941.8	−1003.0
120	(adult abd)*	−87.7	2.9	131.2	944.3	−1000.7
90	(peds abd)*	−85.3	2.1	133.4	931.3	−1008.3
120	(adult head)*	−87.3	2.9	130.3	945.6	−1001.8
90	(peds head)*	−84.4	3.7	134.5	932.3	−1001.8

Asterisks indicate clinical protocols. Listed tolerances only apply to 120 kV for materials other than water and air.
